# Piezoelectric electrospun scaffold incorporating ibuprofen loaded ultrasound-responsive mesoporous silica nanoparticles for tissue regeneration

**DOI:** 10.1039/d5ra05217c

**Published:** 2025-09-17

**Authors:** Alice Benedetto Mas, Jacopo Barberi, Giorgia Montalbano, Silvia Fraterrigo Garofalo, Eleonora Marta, Elsa D. Silva, Rita N. Gomes, Diana S. Nascimento, Sofia Sirolli, Andrea Cafarelli, Sonia Fiorilli, Chiara Vitale-Brovarone

**Affiliations:** a Department of Applied Science and Technology (DISAT), Politecnico di Torino Corso Duca Degli Abruzzi 24 10129 Torino Italy sonia.fiorilli@polito.it; b Consorzio Interuniversitario Nazionale per la Scienza e Tecnologia dei Materiali (INSTM, UdR PoliTO) Italy; c i3S – Institute for Research and Innovation in Health, University of Porto Porto Portugal dsn@i3s.up.pt; d ICBAS – Instituto de Ciências Biomédicas Abel Salazar, University of Porto Porto Portugal; e INEB – Instituto Nacional de Engenharia Biomédica, University of Porto Porto Portugal; f The BioRobotics Institute, Scuola Superiore Sant'Anna Piazza Martiri della Libertà 33 56127 Pisa Italy; g Department of Excellence in Robotics & AI, Scuola Superiore Sant'Anna Piazza Martiri della Libertà 33 56127 Pisa Italy

## Abstract

Inflammation plays a pivotal role in tissue repair, and its modulation is crucial to avoid excessive immune activation or suppression. Three-dimensional scaffolds that can control the release of anti-inflammatory agents over time and space offer a promising approach to regulate the immune response while providing structural support during healing. In this study, we developed an electrospun biomimetic scaffold incorporating ibuprofen-loaded mesoporous silica nanoparticles with an ultrasound-responsive alginate coating. Optimal parameters for safe and non-invasive ultrasound stimulation (*i.e.*, frequency, intensity, and duration) were identified to induce weakening of the interaction among the alginate chains, enabling on-demand drug release from the nanoparticles. *In vitro* studies with human macrophages confirmed the biocompatibility and anti-inflammatory efficacy of the nanoparticles at concentrations up to 1 mg mL^−1^. The results demonstrated that ultrasound stimulation further amplified the therapeutic effect even at very low concentrations (≥0.05 mg mL^−1^) of the tested nanoparticle suspensions. The electrospinning process was optimised to produce nanoparticle-containing polyvinylidene fluoride nanofibers that are aligned and piezoelectric, mimicking the architecture and electroactivity of native tissues. The resulting scaffold exhibited excellent biocompatibility and effectively reduced inflammatory markers *in vitro*. Furthermore, controlled ibuprofen release from the scaffold was successfully triggered on-demand through repeated ultrasound stimulations, applied up to seven days after immersion. By combining structural support, biocompatibility, and the capacity for drug release in response to safe, non-invasive ultrasound stimulations, this scaffold represents a compelling platform for localised modulation of inflammation and the promotion of functional tissue regeneration.

## Abstract 2

The nanoparticle-containing scaffold with aligned electroactive fibers demonstrated anti-inflammatory activity and enabled controlled ibuprofen release upon ultrasound stimulation.

## Graphical abstract

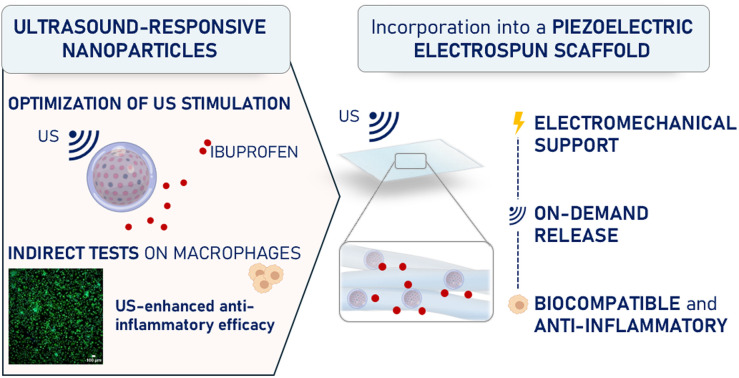

## 1. Introduction

The use of biomaterial-based scaffolds in regenerative medicine offers a valuable alternative to cell-based solutions.^1^ Amongst 3D scaffolds, nanofibrous electrospun membranes are capable of mimicking the structure and orientation of the extracellular matrix (ECM), supporting important features of the healing process such as cell adhesion, proliferation and differentiation.^2^ This can be particularly advantageous for tissues with aligned ECM (*e.g.* tendons, ligaments, myocardium).^3^

The versatility of the electrospinning technique enables the design of multifunctional 3D scaffolds for local drug delivery, either by integrating therapeutic agents directly into the fibers or by embedding drug-loaded nanoparticles within the fibrous architecture. These scaffolds not only support cell attachment and proliferation but also facilitate *in situ* drug release, which is particularly advantageous for minimising systemic toxicity and addressing challenges related to poor pharmacokinetics or non-specific biodistribution of certain drugs.

Considering that the induction of functional tissue recovery after injury or disease requires the regulation of a complex interplay of biological events, achieving spatiotemporal control of local drug release may offer unquestionable benefits.^4–6^ To this end, stimuli-responsive scaffolds enriched with nanocarriers are being developed to enable on-demand drug delivery directly at the disease site.^7–10^ This targeted release can be triggered by either endogenous or external stimuli,^2,11–13^ offering a promising strategy for precise therapeutic intervention. In this context, 3D nanofiber scaffolds, characterised by high surface area and porosity, provide a substantially faster response to stimuli^2^ compared to dense structures, where extensive drug diffusion within the matrix delays the stimulation response.

Given the bioelectrical activities across various human tissues (*e.g.* bone, dentin, tendon, heart, ligaments, cartilage, skin), interest in piezoelectric scaffolds has surged in recent years.^14–16^ By mimicking natural bioelectrical fields generated under mechanical stress, both endogenous and exogenous (*e.g.*, ultrasound stimulation^17^), the scaffold promotes intimate mechanoelectrical interactions with cells, thereby enhancing tissue regeneration.

Among the various nanocarriers used in biomedical applications, mesoporous silica nanoparticles (MSNs) have been extensively studied due to their large pore volume, high specific surface area, low toxicity, and biodegradability.^18^ The uniform and tunable pore size, combined with the ease of surface functionalization, makes them ideal for encapsulating a wide range of molecules.^19–21^ However, the rapid and uncontrolled diffusion of molecules from the nanopores remains a challenge.^22,23^ The undesired passive release can be mitigated by grafting capping layers onto the pore entrances, effectively entrapping therapeutic agents within the mesopores. These capping layers can be engineered to enable the controlled, site-specific release of therapeutics in response to various physical, chemical or biological stimuli.^24–28^ Among the most promising triggering strategies, ultrasound (US) stands out for its ability to penetrate tissues deeply and non-invasively. The accurate selection of stimulation wave parameters (*i.e.*, frequency, intensity, duty cycle, pulse repetition frequency and application time) is key for the safe and effective clinical use of US. The mechanical effect of low-intensity pulsed ultrasound (LIPUS) can be used to trigger drug delivery while enhancing cell proliferation and neovascularisation, along with a mild thermal effect. In the literature, US stimulation has been reported to induce covalent bond cleavage, such as disulfide (S–S) bond splitting^29^ and ring-opening reactions,^30^ as well as disrupt inter- and intramolecular interactions.^31^ Overall, these mechanisms can be exploited to trigger molecular modifications.^32^ While most research on US-responsive nanocarriers has focused on organic particles,^33–37^ studies by the Vallet-Regí group have demonstrated that MSNs functionalized with US-responsive copolymer gatekeepers can enable remotely triggered drug release, effectively preventing premature delivery of cytotoxic agents in cancer therapy.^22,23^

In regenerative tissue research, recent studies have introduced the concept of pro-regenerative inflammation, highlighting the importance of carefully controlling the timing and dosage of anti-inflammatory drug administration.^38–40^ This approach aims to modulate inflammation without hindering its essential role in tissue repair and regeneration. The challenge of tackling the complex progression of regeneration processes, especially in compromised clinical scenarios, inspired us to engineer smart 3D scaffolds by incorporating ultrasound-responsive nanocarriers, which enable the controlled spatiotemporal release of anti-inflammatory drugs.

Building on the elements discussed above, this study comprises for the first time the development, characterisation and proof-of-concept efficacy/biocompatibility of a polyvinylidene fluoride (PVDF)-based piezoelectric electrospun scaffold incorporating US-responsive ibuprofen-loaded MSNs. The primary objective of the developed device is to provide electromechanical support to diseased tissue while enabling precise spatiotemporal control of localised anti-inflammatory drug release, thereby facilitating effective modulation of inflammatory responses.

**Fig. 1:**
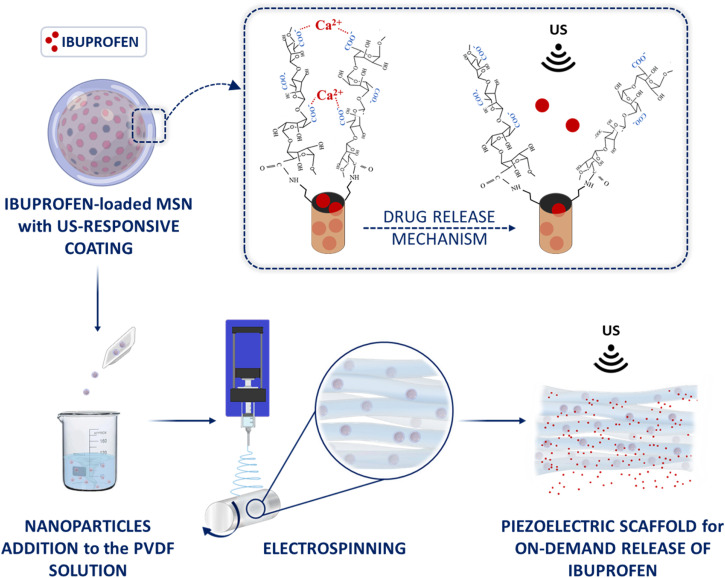
Concept of the study.

Fig. 1 illustrates the overall concept underlying this study, where a polyvinylidene fluoride (PVDF) solution containing US-responsive MSNs is processed by electrospinning (ESP) to manufacture a fibrous composite 3D scaffold.

To impart ultrasound responsiveness to the nanoparticles, the surface of ibuprofen-loaded MSNs was modified with an alginate coating. This biopolymer was selected not only for its extensive use in tissue regeneration^41,42^ but also for its ability to undergo reversible structural changes upon ultrasound stimulation,^43^ thereby conferring the desired responsiveness to the system. By leveraging the mechanical effect of controlled LIPUS stimulation, the ionic interactions between Ca^2+^ ions and the carboxylate groups of the alginate coating are reversibly weakened, enabling ibuprofen to diffuse throughout the nanopores.

## 2. Materials and methods

### 2.1. Materials

For the preparation of the nanoparticles, cetyltrimethylammonium bromide (CTAB, ≥98%), sodium hydroxide (NaOH, ≥98%), tetraethyl orthosilicate (TEOS, reagent grade 98%), ethanol (≥99.8%), 3-(aminopropyl)silanetriol (APST, 20–25% in water), ibuprofen (>98%), hydrochloric acid (ACS reagent, 37%), *N*-(3-dimethylaminopropyl)-*N*′-ethylcarbodiimide hydrochloride (EDC, ≥98%), *N*-hydroxysuccinimide (NHS, 98%), alginic acid sodium salt powder and calcium chloride anhydrous (CaCl_2_, 99.99%) were all purchased from Sigma-Aldrich (St. Louis, USA). For the electrospinning polymeric formulation, PVDF (average MW ≈ 180 kDa), and acetone (ACE) were purchased from Sigma-Aldrich (St. Louis, USA), and dimethyl sulfoxide (DMSO, Uvasol®) from Merck KGaA (Darmstadt, Germany).

### 2.2. Synthesis of US-responsive MSN loaded with ibuprofen (IBU@MSN-SA-Ca)

MSNs were obtained using a modified Stober method, adding a mixture of TEOS and ethanol to an aqueous solution of the structure-directing agent (CTAB) in basic conditions.^44^ The organic template was removed by calcination (550 °C for 6 h, heating rate 1 °C min^−1^) in a Carbolite 1300 CWF 15/5. Amino functionalization of MSN was conducted to graft covalently alginate through the formation of an amide bond. To prepare MSN-NH_2_, the obtained MSN were outgassed at room temperature and grafted with APST under a nitrogen atmosphere using the Schlenk line, as described elsewhere by the authors.^45^ Ibuprofen (IBU) was loaded into MSN-NH_2_ by incipient wetness impregnation. Briefly, aliquots of 100 μL of a concentrated solution of ibuprofen in ethanol (30 mg mL^−1^) were repeatedly dropped onto MSN-NH_2_ powders. Each impregnation step was followed by heating the powders at 60 °C for 10 minutes to allow solvent evaporation. Before proceeding with the subsequent impregnation step, the dried powders were accurately remixed. Impregnation–evaporation steps were repeated to load a theoretical amount of 360 mg IBU per g MSN-NH_2_; the resulting ibuprofen-loaded nanoparticles are referred to as IBU@MSN-NH_2_.

To graft sodium alginate (SA) on IBU@MSN-NH_2_, an aqueous solution of alginic acid sodium salt (at different concentrations of 1%, 2% and 4% wt/vol) was adjusted to pH 5 by adding 1 M HCl. To promote the amide-bond formation, 48 mg of EDC were added to 3.3 mL of SA solution under magnetic stirring. After 30 minutes, 100 mg of IBU@MSN-NH_2_ were slowly added to the solution. Subsequently, 28 mg of NHS were added to the suspension, which was stirred for 2 hours at room temperature. The nanoparticles grafted with SA, named IBU@MSN-SA, were recovered by centrifugation (10 000 rpm, 5 min), washed 3 times with dH_2_O and dried at 70 °C overnight. The alginate coating on the nanoparticles was then crosslinked by adding 100 mg of IBU@MSN-SA to 10 mL of 3% wt/vol CaCl_2_ aqueous solution under magnetic stirring. The suspension was stirred for 30 minutes at room temperature. The resulting nanoparticles (IBU@MSN-SA-Ca) were recovered by centrifugation (10 000 rpm, 5 min), dried overnight at 70 °C and characterised as reported in Section 2.3.

### 2.3. Characterization of the nanoparticles

Nanoparticle morphology after various preparation stages was analysed by Field-Emission Scanning Electron Microscopy (FESEM; Zeiss Merlin instrument, Oberkochen, Germany) upon platinum sputter coating. Transmission electron microscopy (TEM) was conducted on the coated nanoparticles without drug (MSN-SA-Ca), with a Talos F200X instrument (Thermo Scientific) working at 200 kV. N_2_ adsorption–desorption isotherm analyses were performed on outgassed samples using an ASAP2020 PLUS Micromeritics analyser at a temperature of −196 °C. Bare MSN samples were outgassed for 3 hours at 150 °C, while samples loaded with IBU or coated with alginate were outgassed for 3 hours at 100 °C to avoid damage to the drug or coating. Brunauer–Emmett–Teller (BET) equation was used to evaluate the specific surface area (SSA_BET_), while the pore size distribution was calculated through the Density Functional Theory (DFT) model. X-ray diffraction (XRD), performed with a Philips X'Pert diffractometer equipped with a Cu K-α source (40 kV, 40 mA) over a 2*θ* range of 10–80°, was used to confirm the amorphous state of the drug loaded into IBU@MSN-NH_2_. Fourier Transform Infrared Spectroscopy (FTIR, Equinox 55 spectrometer, Bruker, Ettlingen, Germany), carried out in transmission mode on a degassed sample pellet, allowed for the investigation of the anchored functionalities at the nanoparticle surface. The analyses were carried out on nanoparticles without the drug to avoid the overlapping between the signals of the alginate chains and the ibuprofen molecule. The hydrodynamic size (based on number-weighted size distribution) and zeta potential of the nanoparticles were measured in aqueous suspensions (0.1 mg mL^−1^ in deionized water) using a Zetasizer Nano ZS90 instrument (Malvern Instruments Ltd, Malvern, UK). Thermogravimetric analysis (TGA) was carried out under 50 mL min^−1^ air flux, with temperature ramp of 8 °C min^−1^, between 25–1000 °C (25–600 °C for the optimization of alginate grafting concentration), using a TG 209 F1 Libra instrument (NETZSCH GmbH & Co. Holding KG, Selb, Germany). TGA was used to quantify the drug loading, calculated in the range 220–800 °C as the difference in weight loss between IBU@MSN-SA-Ca and MSN-SA-Ca.

For drug release tests, 10 mg mL^−1^ suspensions of nanoparticles in Trizma base 0.1 M (Trizma® pre-set crystals, BioPerformance certified, pH 7.4, Sigma-Aldrich) were prepared and left in the orbital shaker (Excella E24, Eppendorf, Germany) at 37 °C, 100 rpm. At each time step, suspensions were centrifuged (10 000 rpm, 5 min) and supernatants were collected and analysed, while fresh medium was added to resuspend the powders. The supernatants were then diluted with ethanol (50 : 50 in volume) and analysed through High-Performance Liquid Chromatography (HPLC). Analyses were performed using a Shimadzu Nexera (Kyoto, Japan) UV/Vis HPLC system equipped with a core–shell C18 column (Kinetex C18; 150 mm × 4.6 mm, 2.6 μm particle size), an isocratic pump, and an auto-sampler. The experiments were carried out at 30 °C. The mobile phase consisted of a mixture of water (adjusted to pH 2.5 with phosphoric acid, LC grade, Sigma-Aldrich) and acetonitrile (LC grade, Sigma-Aldrich) in a 40 : 60 v/v ratio, delivered at a flow rate of 1 mL min^−1^. Detection was performed using a UV detector at a wavelength of 220 nm. The method was adapted from a previously reported procedure with minor modifications.^46^ A calibration curve was constructed to ensure the accuracy and linearity of the method. The release tests were conducted in triplicate.

### 2.4. Manufacturing and characterisation of PVDF scaffold containing IBU@MSN-SA-Ca

An anisotropic composite scaffold was produced by ESP using a formulation based on PVDF in a DMSO : ACE mixture that contained suspended IBU@MSN-SA-Ca nanoparticles. Briefly, PVDF pellets were dissolved in DMSO : ACE (50 : 50 vol) at a concentration of 18% wt/vol for 4 hours at 50 °C.^47^ Different concentrations of IBU@MSN-SA-Ca nanoparticles were tested, namely 1, 5, 7.5, and 10% wt/vol, to identify the maximum amount that could be incorporated into the fibres without affecting either the ESP process or the scaffold properties (termed PVDF_MSNX_IBU, where X corresponds to nanoparticle concentration in the formulation as % wt/vol). Nanoparticles were slowly added to the fully dissolved PVDF solution while under vigorous stirring to avoid aggregation. ESP was performed on an LP-50 equipment (BIONICIA, Valencia, Spain) featuring a rotating drum collector and a spinneret with a blunt metallic needle (22 G, inner diameter: 0.413 mm, or 16 G, inner diameter: 1.194 mm). The processing parameters were as follows: a voltage of 20 kV, a spinneret-to-collector distance of 12 cm, a flow rate ranging from 1.0 to 1.4 mL h^−1^, and a drum speed of 2000 rpm. Screening tests at different nanoparticle concentrations consisted of 15-minute ESP processes, and samples were evaluated in terms of morphology and crystalline structure, as described below. Further studies focused on formulation processability by increasing the ESP time up to 3 h. After optimization, selected PVDF_MSN5_IBU fibres were deposited for 3 h, resulting in membranes with a thickness between 100 and 150 μm. These samples were fully characterized as follows.

The morphology of the scaffolds was observed by FESEM (Zeiss Merlin, Zeiss, Oberkochen, Germany), and the diameter was measured with the ImageJ plug-in DiameterJ.^48^ PVDF exists in three primary isomorphic forms: α, β, and γ. Among these, only the β phase, characterized by its all-trans planar structure, exhibits piezoelectric properties, consequently, the piezoelectric activity of PVDF is contingent upon the relative amount of β phase. The amount of the crystalline α, β, and γ phases was determined by FTIR in attenuated total reflection (ATR) mode, according to the equations reported by Cai *et al.*^49^ The total crystallinity (*X*_c_) was obtained by differential scanning calorimetry (DSC; Mettler Toledo DSC-1, Columbus, USA), heating the samples from 30 °C to 200 °C at 10 °C min^−1^ under N_2_ flow. *X*_c_ was evaluated according to the equation reported by He *et al.*,^50^ taking as melting enthalpy of the different crystalline phases 103.4 J g^−1^ for both α and γ phases,^51^ and 93.07 J g^−1^ for the β phase.^49^ The piezoelectric coefficient *d*_33_ was measured using a piezo evaluation system (PES; TF Analyzer 2000HS, Aixacct, Aachen, Germany) and a single-point laser vibrometer (Polytec OVDF-505, Waldbronn, Germany) by collecting voltage-displacement hysteresis curve at a frequency between 1–10 kHz. The *d*_33_ coefficient was estimated by interpolating the linear segment of these curves. Before measurements, Pt electrodes were sputter-coated onto the samples.

The mechanical properties of the composite membrane were evaluated through tensile tests in accordance with ISO standard 527-5A.^52^ Dog-bone-shaped samples were punctured from the membrane along the fibre direction and tested using an Instron 5966 instrument (50 N load cell). The clamps were positioned 5 cm apart, and tests were conducted at a speed of 10 mm min^−1^ until rupture, with a pre-load of 0.1 N. Five specimens were tested.

For drug release experiments without US stimulation, 30 mg of PVDF_MSN5_IBU were immersed in 1 mL of 0.1 M Trizma base and kept in an orbital shaker at 37 °C and 100 rpm. At each time interval, supernatants were collected, and fresh medium was replenished in the samples. The supernatants were subsequently prepared for HPLC analysis, following the protocol outlined in Section 2.3. Before drug release studies, PVDF_MSN5_IBU membranes were treated with low-pressure oxygen plasma (100% O_2_, 180 s, 45 W; FEMTO, Diener electronic, Ebhausen, Germany) to enhance the hydrophilicity and cell response of the electrospun scaffolds, according to a protocol previously optimised by the authors.^53^

### 2.5. Ultrasound stimulation setup and parameters

**Fig. 2:**
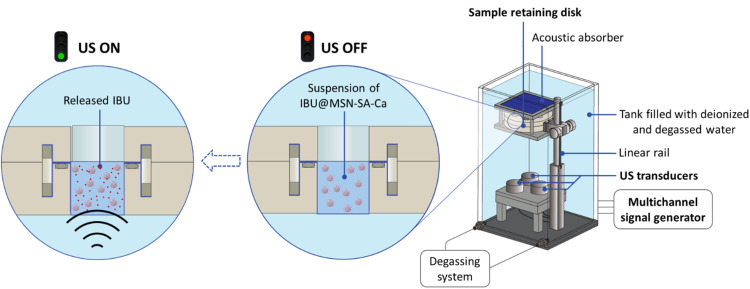
High-frequency US stimulation setup.

Two *ad hoc* systems for Low Intensity Pulsed US (LIPUS) stimulation were employed: one to cover the range of low-frequency stimulation (38 kHz) and the other for high frequencies (2 MHz and 5 MHz). These patented systems^54^ were specifically designed by the authors to avoid uncontrolled propagation phenomena in the acoustic path from transducer to target.^55,56^ Therefore, they allow a high control over the US dose reaching the stimulated samples, enhancing the reliability and repeatability of the results. Both systems have the same basic structure, with slight differences due to frequency constraints. As displayed in Fig. 2, the high-frequency system is equipped with a tank filled with deionised and degassed water to avoid the presence of gas bubbles in the acoustic path. An acoustic absorber, placed at the top of the tank, avoids reflections of the US wave. The sample holder consists of a US-transparent disk with 3 chambers sealed with a Stretchlon membrane, guaranteeing waterproof sealing and transparency to US waves. Three fully characterised piezoceramic transducers (Precision Acoustics, Dorchester, Dorset, UK), centred at the chosen operative frequencies (2 MHz, 5 MHz), are driven by a multichannel signal generator (Image Guided Therapy, Bordeaux, France), allowing the stimulation of 3 samples in parallel. A linear rail allows for regulating the distance between the sample holder and the transducers, so that the samples are in the focus of the US field at any frequency. The low-frequency setup is equally structured, but it accommodates only one transducer, centred at 38 kHz (BAC Technology, Florence, Italy) and driven by a dedicated signal generator (SIRIO, BAC Technology, Florence, Italy). Therefore, the sample retaining disk only has one chamber, aligned with the transducer, allowing the stimulation of one sample at a time.

Firstly, the release of ibuprofen upon ultrasound (US) stimulation of IBU@MSN-SA-Ca was tested by preparing a nanoparticle suspension (10 mg mL^−1^) in 0.1 M Trizma base. To better distinguish the effect of US stimulation in triggering the release of loaded ibuprofen, the nanoparticles were pre-conditioned in the medium to promote diffusion-based release, which is particularly prominent during the initial hours after soaking. To this purpose, the suspension was incubated in an orbital shaker at 37 °C, 100 rpm for 48 hours and subsequently centrifuged (10 000 rpm, 5 min), discarding the supernatant. The recovered pre-conditioned nanoparticles were resuspended in fresh medium, transferred into the sample holder and exposed to US stimulation (Fig. 2). After US stimulation, the suspension was centrifuged, and the supernatant was recovered for HPLC analysis.

Fresh medium was added to the powders, and the suspension was incubated (37 °C, 100 rpm) until the second US stimulation, applied 24 hours later. Tests were conducted in triplicate for each stimulation condition investigated.

Similarly, PVDF_MSN5_IBU scaffold (45 mg) was soaked in 1.5 mL of 0.1 M Trizma base at 37 °C for 48 h prior to US stimulation. After pre-conditioning, the supernatant was removed and the membrane transferred into the sample holder, where fresh medium was added to achieve a PVDF_MSN5_IBU concentration of 30 mg mL^−1^. After US stimulation, the supernatant was collected and analysed by HPLC. The membrane was subsequently immersed in fresh medium and incubated at 37 °C and 100 rpm until the next stimulation. Alongside the second US stimulation applied 24 h after the first, the effect of a third US stimulation, 5 days after the first one, was analysed. All the tests were performed in triplicate.

### 2.6. Isolation and culture of human macrophages (hMACs)

Human samples were obtained in agreement with the principles of the Declaration of Helsinki. Peripheral Blood Mononuclear Cells (PBMCs) were isolated from surplus buffy coats, kindly provided by the Immunohemotherapy Department of Centro Hospitalar São João (CHSJ), Porto, Portugal.

The procedures were approved by the Hospital and the i3S Ethical Committee.^57^ Informed written consent that the by-products of their blood collections could be used for research purposes was obtained from the blood donors and their identity was archived exclusively at CHSJ. Primary hMACs were differentiated from monocytes obtained from buffy coats using the RosetteSep™ human monocyte enrichment antibody cocktail (STEMCELL Technologies), following established protocols.^58,59^ Isolated monocytes were plated on low-attachment plates (270 000 cells per cm^2^) in medium hMAC—consisting of RPMI 1640 + Glutamax (Invitrogen), with 10% heat-inactivated Fetal Bovine Serum (FBS, Biowest) and 1% Penicillin/Streptavidin (P/S, Gibco)—supplemented with macrophages colony stimulating factor (M-CSF, 50 ng mL^−1^, Immunotools). Cells were maintained at 37 °C with 5% CO_2_ for 7 days, without media change or plate manipulation.

### 2.7. Dose response to ibuprofen

hMACs were washed with PBS and cultured in (i) medium hMAC (CTR, non-stimulated condition), (ii) medium hMAC with 10 ng mL^−1^ lipopolysaccharide (LPS, stimulated condition) or (iii) medium hMAC with 10 ng mL^−1^ LPS and ibuprofen at different concentrations (15, 30, 60, 200, and 600 μg mL^−1^). hMACs were maintained for 3 days at 37 °C with 5% CO_2_.

### 2.8. *In vitro* biocompatibility and efficacy assessment

Before all cell culture experiments, the nanoparticles and the composite scaffold with and without ibuprofen (IBU@MSN-SA-Ca and MSN-SA-Ca; PVDF_MSN5_IBU and PVDF_MSN5) were UV-sterilised for 30 minutes.

#### 2.8.1 Direct assay on the nanoparticles

To test the biocompatibility and efficacy of IBU@MSN-SA-Ca and MSN-SA-Ca nanoparticles when in direct contact with cells, hMACs were washed with PBS and cultured in medium hMAC with 10 ng mL^−1^ of LPS and containing 0.1 or 1 mg mL^−1^ of either IBU@MSN-SA-Ca or MSN-SA-Ca nanoparticles. Cells were maintained at 37 °C with 5% CO_2_ for 3 days. Four technical replicates were performed per condition, and the CTR and LPS controls were maintained through the experiment.

#### 2.8.2 Indirect assay on the nanoparticles and on the composite scaffold

To test the biocompatibility and efficacy of the components released by the IBU@MSN-SA-Ca and MSN-SA-Ca, 1.1 mg mL^−1^ of nanoparticles were suspended and incubated (37 °C) with medium hMAC without FBS at different time points, as illustrated in Fig. S1a. The nanoparticles were initially suspended at 1.1 mg mL^−1^ so that, upon addition of 10% v/v FBS to the medium before incubation with hMACs, the final nanoparticle concentration was equal to 1 mg mL^−1^. For the indirect assay on the scaffold, PVDF_MSN5 and PVDF_MSN5_IBU were punched into circular discs (12 mm in diameter) and incubated at 37 °C in 10 mL of hMAC medium without FBS.

In both experiments, the objective was to obtain and collect the components released by the nanoparticles and from scaffolds during the first 24 h (D1), from 24 h to 48 h (D2) and during day 4 and day 5 (D4–5) of incubation in a coordinated manner so the media was tested immediately following collection, avoiding unnecessary storage which could impact on the biological activity of samples. The collected media was centrifuged at 4000 rpm for 5 minutes to eliminate the nanoparticles and any residual scaffold fragments. For experiments involving nanoparticles, the supernatant was supplemented with 10% v/v FBS and either applied directly to hMACs (corresponding to nanoparticle concentration of 1 mg mL^−1^) or diluted 10-fold in medium hMAC (0.1 mg mL^−1^). For scaffold-derived media, the supernatant was similarly supplemented with 10% v/v FBS and applied directly to cells, corresponding to a scaffold concentration of 0.1 cm^2^ mL^−1^. hMACs were washed with PBS and cultured at 37 °C with 5% CO_2_ for 3 days in the media collected from either nanoparticles or scaffold samples, in the presence of 10 ng mL^−1^ of LPS. Three technical replicates were performed for each tested condition, and the CTR and LPS controls were maintained through the experiment.

#### 2.8.3 Ultrasound-stimulated release from IBU@MSN-SA-Ca

IBU@MSN-SA-Ca were resuspended (0.11 mg mL^−1^) in medium hMAC without FBS and stimulated with US (2 MHz frequency, 2000 mW cm^−2^ intensity, 1 kHz pulse repetition frequency, 20% duty cycle, for 3 min) using the setup described in Section 2.5 (Fig. S1b). After 24 h incubation at 37 °C with 5% CO_2_, samples were centrifuged (4000 rpm, 5 min) to separate the nanoparticles from the media. The supernatant was then supplemented with 10% v/v FBS: the resulting media, corresponding to a nanoparticle concentration of 0.1 mg mL^−1^, was either incubated with hMACs or diluted to concentrations of 0.05 and 0.025 mg mL^−1^ before incubation. hMACs were washed with PBS and cultured at 37 °C with 5% CO_2_ for 3 days in the media collected from nanoparticles in the presence of 10 ng mL^−1^ of LPS. Three technical replicates were performed per condition, and the CTR and LPS controls were maintained through the experiment.

#### 2.8.4 Metabolic activity assay

hMACs were incubated for 80 min in medium hMAC containing 10% of resazurin (Sigma-Aldrich, St. Louis, MO, USA) at 37 °C with 5% CO_2_. The media was collected, and fluorescence (*λ*_ex/em_ = 530/590 nm) was measured using a Synergy Mx micro-plate reader spectrophotometer (Bio-Tek Instruments, Winooski, VT, USA). Data were normalised to the LPS condition.

#### 2.8.5 Cell viability assay

hMACs were incubated with Calcein (0.001% v/v in PBS) and Hoechst (0.0005% v/v in PBS) during 20 min and washed twice with PBS. Images were acquired using a high-throughput Operetta CLS confocal microscope (Revvity) and analysed using a bioinformatics pipeline developed in Harmony software.

#### 2.8.6 Assessment of anti-inflammatory activity

Since ibuprofen inhibits cyclooxygenase (COX) enzymes—key mediators of prostaglandin synthesis—its anti-inflammatory activity was assessed by measuring the inhibition of prostaglandin *E*_2_ (PGE_2_) secretion. PGE_2_ levels were quantified using the DetectX ELISA kit (Arbor Assays™, K051-H1/H5) according to the manufacturer's instructions. Absorbance was read at 450 nm (BioTek Gen5).

#### 2.8.7 Statistical analysis

Data were analysed using GraphPad Prism 8.3.0 software. Normally distributed data were tested with a paired Student's *t*-test and one-way ANOVA (Turkey post-hoc test) test for two and three or more groups. The differences between groups are considered significant when *p* < 0.05 (*p < 0.05, **p < 0.01, ***p < 0.001, and ****p < 0.0001).

## 3. Results and discussion

### 3.1. Characterisation of the nanoparticles

FESEM analysis on MSN and MSN-NH_2_ (Fig. S2a and b) revealed spherical nanoparticles with a uniform size of approximately 120 nm and confirmed that the surface modification did not alter the morphology. Hydrodynamic size measurements indicated monodisperse distributions centered at 159 ± 6 nm for MSN and 164 ± 19 nm for MSN-NH_2_. The size overestimation compared to FESEM is consistent with known differences between hydrodynamic and dry-state measurements due to solvation and surface effects.^60^ XRD analysis on IBU@MSN-NH_2_ assessed the amorphous state of IBU, essential to provide an enhanced dissolution rate of the drug.^61^ IBU@MSN-NH_2_ exhibited a monodisperse size distribution, with a hydrodynamic diameter centered at 166 ± 12 nm, showing no significant deviation from that of the unloaded MSN-NH_2_.

This study investigated the grafting of higher sodium alginate concentrations (1%, 2%, and 4% wt/vol) to effectively limit diffusion-driven drug release, thereby improving ibuprofen retention within the nanoparticles.^62,63^ As shown in Fig. S3, TGA analysis indicated that increasing SA concentrations resulted in a greater amount of alginate grafted onto the nanoparticle surface, leading to an increase in weight loss from 12% to 18% within the 220–600 °C range, attributed to alginate degradation.

FESEM analysis confirmed the formation of distinct nanoparticles with spherical morphology across all tested SA concentrations. Based on these results, the highest SA concentration (4% wt/vol) was selected, as increasing the amount of grafted alginate is anticipated to enhance capping efficiency while maintaining nanoparticle morphology.

**Fig. 3:**
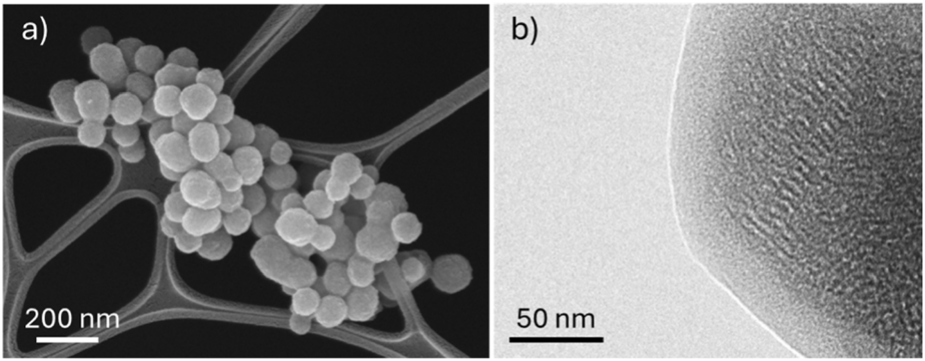
(a) FESEM image of IBU@MSN-SA-Ca and (b) TEM micrography of MSN-SA-Ca.

FESEM image of the crosslinked nanoparticles is reported in Fig. 3a: compared to unfunctionalised samples (Fig. S2a), the size and morphology were not significantly altered by the polymeric capping. This observation was further supported by hydrodynamic diameter measurements of MSN-SA-Ca, which revealed a monodisperse size distribution centered at 170 ± 19 nm. The ordered mesoporous structure of MSN and the homogeneous alginate coating (average thickness 20 nm) can be observed in TEM micrography (Fig. 3b).

**Fig. 4:**
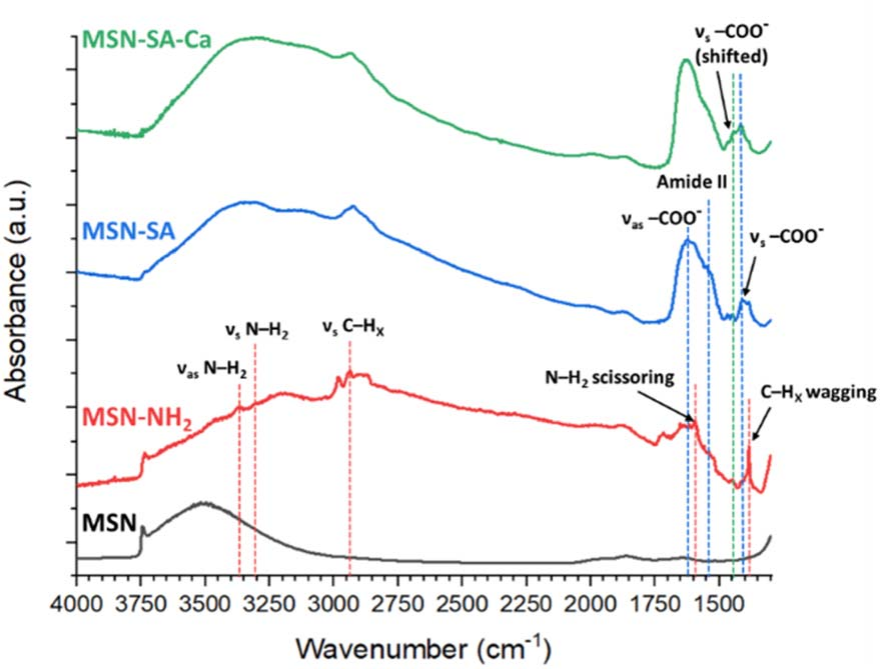
FTIR spectra of bare MSN (black line), MSN-NH_2_ (red), MSN-SA (blue), and MSN-SA-Ca (green).

FTIR spectra of MSNs at various stages of surface modification are reported in Fig. 4. The peak associated with –NH_2_ scissoring (*ca.* 1585 cm^−1^) and two bands in the region of –NH_2_ stretching (3301 cm^−1^ symmetric and 3366 cm^−1^ asymmetric, respectively) were detected in the spectrum of MSN-NH_2_.^64^

–NH_2_ stretching vibration signals are overlapped with the broad band observed at 3000–3600 cm^−1^, assigned to the stretching of hydrogen-bonded O–H groups. Moreover, due to the presence of the alkyl chains of APST, peaks associated with C–H_*x*_ vibrations (wagging at 1384 cm^−1^ and symmetric stretching at 2934 cm^−1^) were identified, confirming the successful surface modification. In the MSN-SA spectrum, successful grafting with sodium alginate was confirmed by the peaks assigned to the symmetric (1413 cm^−1^) and asymmetric (1611 cm^−1^) stretching of the –COO^−^ groups exposed along the alginate chains. Around 1540 cm^−1^, the peak associated with amide II vibration mode confirmed the formation of amide bonds between the amino groups on the silica surface of MSN-NH_2_ and the carboxyl groups of alginate.^65–67^ The spectrum of crosslinked nanoparticles (MSN-SA-Ca) displayed the same peaks detected in the MSN-SA spectrum, along with an additional peak around 1430 cm^−1^. This new signal was attributed to the shift of the peak of COO^−^ symmetrical stretching to a higher wavenumber, resulting from the replacement of sodium ions with calcium ions in the alginate network. The stronger interaction between calcium ions and the carboxylate groups of adjacent alginates accounted for this shift.^67^

Isothermal plots and pore size distributions (DFT) derived from nitrogen adsorption–desorption analyses on MSN, MSN-NH_2_ and MSN-SA-Ca are shown in Fig. S4a and b. The values of BET Specific Surface Area (SSA_BET_), total pore volume and zeta potential are reported in Table 1. MSN with a high specific surface area and a narrow pore size distribution (2.5–4 nm) were obtained. As expected, a reduction in SSA_BET_ and total pore volume was observed after the functionalization of MSN with APST, while pore size distribution shifted to lower values (2–3.5 nm). Compared to MSN, the zeta potential became less negative due to protonated amino groups on silica surface. Nitrogen adsorption–desorption analysis on IBU@MSN-NH_2_ revealed a total pore volume of 0.06 cm^3^ g^−1^. This marked reduction compared to MSN-NH_2_ (0.38 cm^3^ g^−1^) confirmed that the mesopores were effectively filled with ibuprofen. The isothermal plot of MSN-SA-Ca (Fig. S4a) presented a lower amount of adsorbed nitrogen compared to MSN-NH_2_. The significant decrease in specific surface area and pore volume in MSN-SA-Ca indicated that the crosslinked alginate coating effectively capped the MSN nanopores, resulting in negligible porosity below 5 nm (Fig. S4b). MSN-SA-Ca showed a negative zeta potential due to exposed negatively charged carboxylates along the alginate chains (zeta potential of MSN-SA was −17 ± 3 mV), packed upon crosslinking with Ca^2+^. Ibuprofen loading into IBU@MSN-SA-Ca was estimated around 13.5%wt by TGA analysis (Fig. S5). Drug release testing of IBU@MSN-SA-Ca confirmed that alginate crosslinking enhanced ibuprofen retention within the nanoparticles (Fig. S6a), resulting in the release of 44% of the total loaded drug over 7 days, nearly half of the 81% released from non-crosslinked nanoparticles. As a burst release of ibuprofen was observed within the first few hours of nanoparticle immersion in the release medium, the nanoparticles were preconditioned for 48 hours prior to US stimulation, as described in Section 2.5, to reduce passive drug diffusion and enhance the effectiveness of the US-triggered release. Fig. S6b presents the ibuprofen release profile from a 10 mg mL^−1^ suspension of IBU@MSN-SA-Ca, expressed as released drug concentration (μg mL^−1^). These released concentrations were utilised to determine the nanoparticle concentration in the suspensions used for the *in vitro* assays reported in the following sections.

**Table 1:** Specific surface area, pore volume and zeta potential of MSN, MSN-NH_2_ and MSN-SA-Ca

Sample	BET specific surface area	Total pore volume	Zeta potential
MSN	1280 ± 30 m^2^ g^−1^	0.77 cm^3^ g^−1^	– 27 ± 1 mV
MSN-NH_2_	760 ± 16 m^2^ g^−1^	0.38 cm^3^ g^−1^	–14 ± 4 mV
MSN-SA-Ca	40.5 ± 0.3 m^2^ g^−1^	0.07 cm^3^ g^−1^	–10 ± 3 mV

#### 3.1.1 Optimisation of US stimulation parameters on the nanoparticles

An extensive literature review was carried out to identify the US protocols required for inducing the release of therapeutics from various drug delivery systems, including MSNs, polymeric nanoparticles and hydrogels. The survey evidenced a huge variability both in the adopted setup and US parameters: frequency 20 kHz–12 MHz;^62,68^ intensity: 9.6–9020 mW cm^−2^;^43,62^ duty cycle: 10–100%^69^ and stimulation duration: 10 s–120 min (ref. 66 and 70). Additionally, most studies use US setups that do not allow precise control of the applied US energy. Therefore, in this study, the effect of US parameters on the developed nanoparticles was evaluated to identify the optimal US dose for triggering ibuprofen delivery. To minimise potentially harmful temperature increase due to US energy dissipation, intensities <3000 mW cm^−2^ were used, while the duty cycle was fixed at 20% and the pulse repetition frequency at 1 kHz. The US parameters used in the study are reported in Table S1. For all stimulation conditions, temperature increase in the release medium was monitored using a thermocouple placed in the sample holder. In all cases, the temperature rise was <0.6 °C, confirming that ibuprofen release from the nanoparticles was driven by mechanical rather than thermal effect, and ensuring compliance with safety standards.^71^ In this study, the mechanical action of US was exploited to reversibly weaken the electrostatic interactions between carboxylate groups (–COO^−^) and calcium ions (Ca^2+^)^43^ within the alginate coating of IBU@MSN-SA-Ca. This US-triggered destabilisation of the polymeric coating enhances its permeability, thereby facilitating the diffusion of ibuprofen molecules from the mesoporous silica core into the surrounding medium. First, the effect of US frequency was investigated (experiment A), keeping unaltered the rest of the parameters. Once identified 2 MHz as the most efficient frequency in inducing ibuprofen release (Fig. S7a), the frequency was fixed, and a screening of the stimulation intensities was carried out (experiment B, Fig. S7b). Lastly, stimulation duration was prolonged up to 5 minutes (experiment C1), but no further enhancement in ibuprofen release was assessed.

**Fig. 5:**
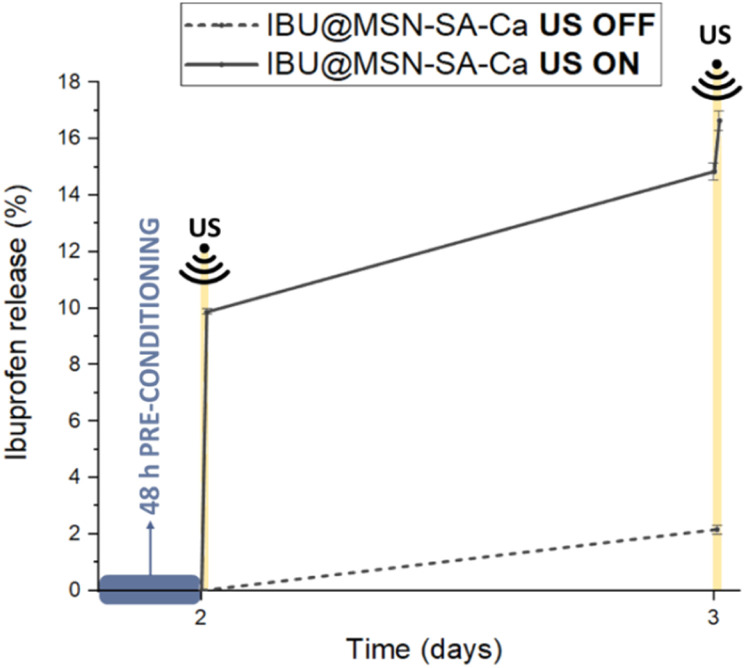
Drug release from IBU@MSN-SA-Ca in the absence of US stimulation (dashed line) and upon optimized US stimulation applied at day 2 and day 3 (solid line).

Among the tested US conditions, frequency =, intensity = 2000 mW cm^−2^, stimulation duration = 3 min (experiment B4) were the most effective in triggering on-demand ibuprofen release, enabling the delivery of 10% of the total loaded drug (Fig. 5). A second US stimulation applied after 24 hours induced additional ibuprofen release (2%). Notably, the optimised stimulation time of 3 minutes is shorter than those reported in previous studies on US-responsive MSN systems (*i.e.* 5 min,^68^ 10 min (ref. 22, 62 and 72) or 20 min (ref. 73)). This reduced activation helps in minimising the risk of thermal damage associated with prolonged US exposure, consequently enhancing the potential for clinical translatability.

### 3.2. *In vitro* cytotoxicity and anti-inflammatory effect of ibuprofen

Due to their pivotal role in orchestrating inflammatory responses, human macrophages (hMACs) were selected as the *in vitro* model for a comprehensive evaluation of the developed ibuprofen-releasing nanoparticles. Human monocytes were collected from the buffy coats of healthy donors and differentiated into macrophages using a standardised assay. Then, to induce an inflammatory condition, hMACs were stimulated with LPS as previously reported,^74^ resulting in a significant average increase by 35-fold in the inflammatory mediator PGE_2_ when compared to non-stimulated hMACs (CTR) (Fig. S8a and b). To determine the therapeutic window in which ibuprofen exerts anti-inflammatory effects without cytotoxicity, a dose–response assay was conducted (Fig. S8c and d). Ibuprofen showed no cytotoxic effects in hMACs at concentrations below 200 μg mL^−1^. However, ibuprofen at 200 μg mL^−1^ and 600 μg mL^−1^ reduced cell viability to 82% and 10%, as measured by calcein^+^ staining (Fig. S8c), indicating minimal and severe toxicity, respectively. Compared to the LPS condition, 15 and 30 μg mL^−1^ of IBU were effective in significantly reducing PGE_2_ secretion by approximately 8- and 11-fold, respectively (Fig. S8d). Based on these findings, the therapeutic window for ibuprofen in this model was defined as 15 to 200 μg mL^−1^.

#### 3.2.1 Biocompatibility of nanoparticles in direct contact with human macrophages

**Fig. 6:**
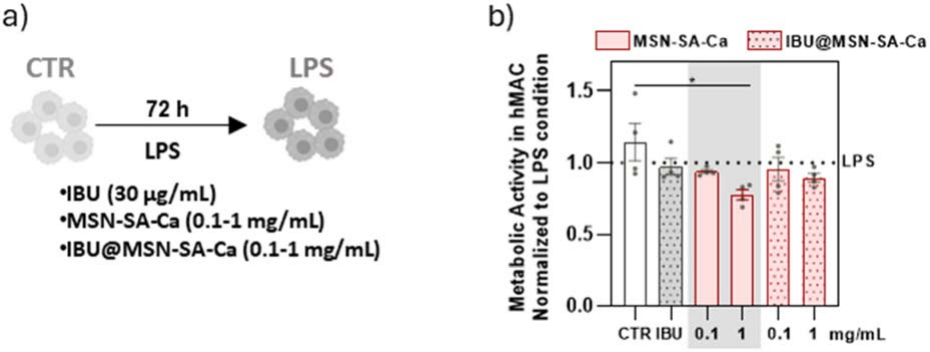
Evaluation of biocompatibility of the MSN-SA-Ca and IBU@MSN-SA-Ca on hMACs. (a) hMACs were stimulated with LPS, either in the presence or absence of IBU, 0.1 mg mL^−1^ and 1 mg mL^−1^ of MSN-SA-Ca and IBU@MSN-SA-Ca, during 72 h. hMACs cultured in the absence of LPS were used as control (CTR). (b) Evaluation of hMAC metabolic activity. Results are normalized to LPS condition (*n* = 4 patients/group). Data show mean ± SEM. For statistical analysis a parametric multiple comparison evaluation through paired one-way ANOVA test was performed to assess differences between groups. Statistical significance is indicated as *p < 0.05, **p < 0.01, ***p < 0.001 and ****p < 0.0001.

A first assessment of MSN-SA-Ca and IBU@MSN-SA-Ca cytocompatibility was performed following direct contact of nanoparticles with hMACs (direct assay). Considering the therapeutic window of ibuprofen (15–200 μg mL^−1^) and its release profile from IBU@MSN-SA-Ca (Fig. S6b), a nanoparticle concentration of 1 mg mL^−1^ was selected, enabling the release of drug in the effective therapeutic range. Accordingly, hMACs were exposed to either 1 mg mL^−1^ or a 10-fold diluted suspension (0.1 mg mL^−1^) of IBU@MSN-SA-Ca or MSN-SA-Ca for 72 hours (Fig. 6a). Metabolic activity assays showed that 0.1 mg mL^−1^ and 1 mg mL^−1^ of IBU@MSN-SA-Ca and MSN-SA-Ca had no or minimal impact, respectively, on cell viability (Fig. 6b).

It is worth noting that the identified cytocompatible concentration (1 mg mL^−1^) is higher than that reported for a 72-hour incubation of MSNs-based nanoparticles with RAW264.7 macrophages (0.02 mg mL^−1^ (ref. 75)), as well as those observed in studies involving polymer-functionalized MSNs on immortalised HeLa cells—0.06 mg mL^−1^ over 24 hours,^76^ and 0.0225 mg mL^−1^ over 48 h.^77^ Moreover, it is important to emphasize that primary cells, such as the hMACs utilized in this study, closely reflect the physiological state of native tissues, providing a more relevant model for studying cellular responses and drug effects compared to immortalized cell lines, which often develop altered signalling pathways, higher resistance to stressors and diminished phenotypic accuracy due to extended adaptation *in vitro*.

#### 3.2.2 Diffusion test of IBU@MSN-SA-Ca biocompatibility and efficacy in human macrophages

**Fig. 7:**
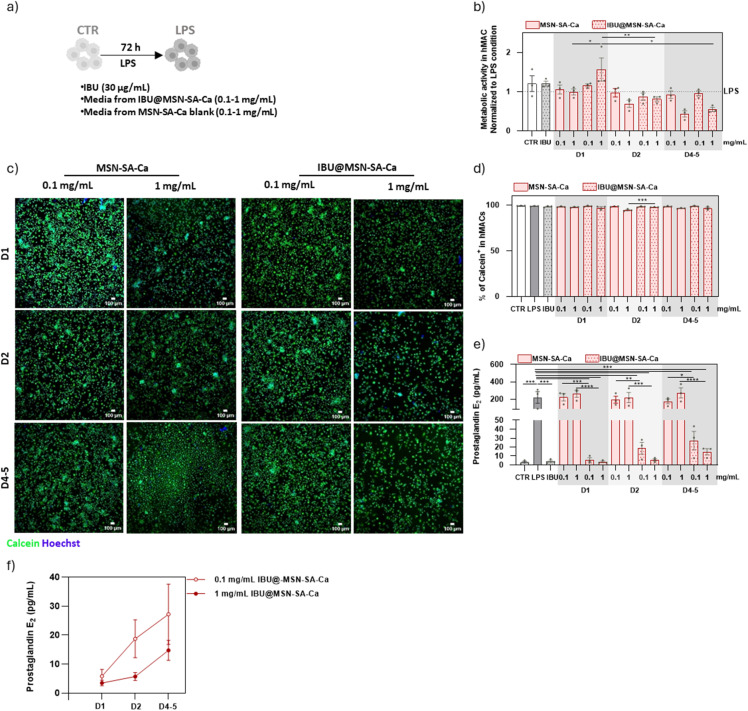
Evaluation of biocompatibility and anti-inflammatory properties of the components released by MSN-SA-Ca and IBU@MSN-SA-Ca on hMACs. (a) hMACs were stimulated with LPS, either in the presence or absence of IBU or the conditioned media released by 0.1 and 1 mg mL^−1^ of MSN-SA-Ca and IBU@MSN-SA-Ca during day 1 (D1), day 2 (D2) and day 4–5 (D4–5), during 72 h. hMACs cultured in the absence of LPS were used as control (CTR). (b) Evaluation of hMAC metabolic activity. Results are normalized to LPS condition (*n* = 4 patients/group). (c) and (d) Representative images (c) and respective quantification (d) of hMAC viability. hMACs were stained with calcein (green) and the nuclei stained with Hoechst (blue). Scale bars = 100 μm (*n* = 4 patients/group). (e) and (f) Quantification of secreted PGE_2_ (pg mL^−1^) by hMAC (*n* = 4 patients/group). Data show mean ± SEM. For statistical analysis a parametric multiple comparison evaluation through paired one-way ANOVA test was performed to assess differences between groups. Statistical significance is indicated as *p < 0.05, **p < 0.01, ***p < 0.001 and ****p < 0.0001.

After characterising the behaviour of IBU@MSN-SA-Ca in direct contact with hMACs, the focus shifted to an indirect assay, which more closely mimics the drug release conditions expected after nanoparticle incorporation into the PVDF fibrous scaffold. As illustrated in Fig. S1a, we analysed the components released from 0.1 and 1 mg mL^−1^ of nanoparticles during day 1 (D1), day 2 (D2) and from day 4 to 5 (D4–5). The data obtained from metabolic activity assays and calcein^+^ cell quantification provide compelling evidence regarding the biocompatibility of IBU@MSN-SA-Ca nanoparticles (Fig. 7a and b). Although treatment with 1 mg per mL IBU@MSN-SA-Ca resulted in a modest reduction in metabolic activity at days 4–5 (Fig. 7a and b), no significant differences were observed in the number of calcein^+^ cells across all experimental groups and time points (Fig. 7c and d). These findings strongly support that components released by IBU@MSN-SA-Ca and MSN-SA-Ca nanoparticles are cytocompatible throughout the duration of the study. Importantly, the release of IBU from IBU@MSN-SA-Ca was found to markedly suppress PGE_2_ levels (Fig. 7e), unequivocally demonstrating the anti-inflammatory efficacy of IBU-loaded nanoparticles, at variance with MSN-SA-Ca, which, as expected, had no impact on PGE_2_, compared to LPS condition (Fig. 7e). Considering D4–5, a period in which the release of IBU concentration is below 15 μg mL^−1^ for both nanoparticle concentrations (Fig. S6b), anti-inflammatory activity was significantly detected (Fig. 7e). These findings align with previous studies reporting an enhanced pharmacological effect of ibuprofen when encapsulated in silica nanocarriers, compared to its soluble form.^78^ Regarding temporal efficacy, the anti-inflammatory effect of IBU@MSN-SA-Ca was more pronounced on D1 and D2 compared to D4–5 (Fig. 7f), as the IBU released from the nanoparticles resulted in the highest concentration following soaking in the medium. Although a significant difference between nanoparticle doses was observed only on day 2, with 1 mg mL^−1^ inducing lower PGE_2_ levels (Fig. 7f), the overall reduction in PGE_2_ confirmed that ibuprofen retained its bioactivity following nanoparticle encapsulation.

#### 3.2.3 Anti-inflammatory activity of US-stimulated IBU@MSN-SA-Ca

**Fig. 8:**
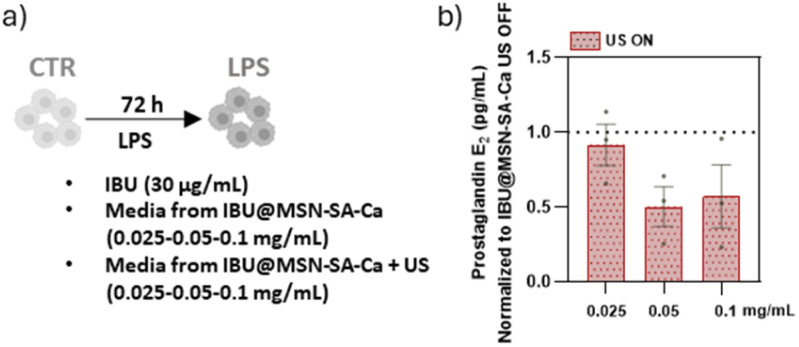
Impact of US stimulation on the anti-inflammatory activity of IBU@MSN-SA-Ca on hMACs. (a) hMACs were stimulated with LPS, either in the presence or absence media released by 0.025, 0.05 and 0.1 mg mL^−1^ of IBU@MSN-SA-Ca during day 1 (D1) with or without US stimulation, during 72 h. hMACs cultured in the absence of LPS were used as control. (b) Quantification of secreted PGE_2_ (pg mL^−1^) by hMAC (*n* = 4 patients/group). Results are normalized to the PGE_2_ levels secreted by hMACs exposed to media from the respective IBU@MSN-SA-Ca concentration without US stimulation (*n* = 3 patients/group). Data show mean ± SEM.

To gain a comprehensive understanding of the behaviour of IBU@MSN-SA-Ca nanoparticles, the final stage of evaluation involved an indirect assay to assess drug release triggered by US stimulation and to evaluate the resulting anti-inflammatory effect. Specifically, released extracts from 0.025, 0.05 and 0.1 mg mL^−1^ of IBU@MSN-SA-Ca over 24 h (D1), either with or without US stimulation, were analysed. The rationale for testing doses below 0.1 mg mL^−1^ was to simulate lower-dose therapeutic scenarios and assess whether US-triggered release would remain effective under these conditions (Fig. 8a). Fig. 8b shows the quantification of PGE_2_ secreted by hMACs exposed to media released from nanoparticles following US stimulation, normalised to PGE_2_ levels from cells exposed to media from the corresponding concentrations of non-stimulated particles. Notably, US stimulation resulted in a dose-dependent reduction of PGE_2_ levels compared to non-triggered nanoparticles, leading to a two-fold enhancement in anti-inflammatory activity at nanoparticle concentrations ≥0.05 mg mL^−1^ (Fig. 8b). This confirmed that the US effectively triggers IBU release from IBU@MSN-SA-Ca, providing therapeutic efficacy even at very low doses.

These findings highlight the potential of US-responsive IBU@MSN-SA-Ca nanoparticles for on-demand anti-inflammatory therapy, in scenarios requiring localised and temporally controlled modulation of inflammation.

### 3.3. Optimisation and characterisation of the electrospun composite scaffold

**Fig. 9:**
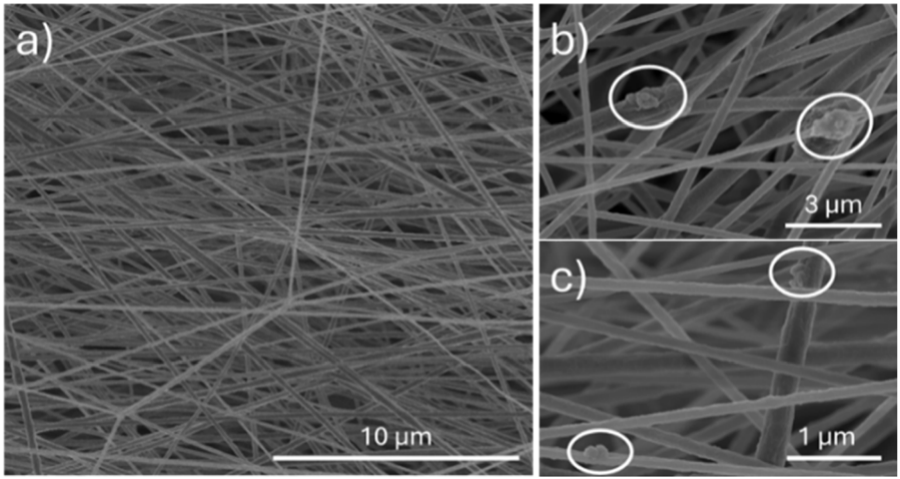
(a) FESEM image of PVDF_MSN5_IBU; details of IBU@MSN-SA-Ca within the fibers in small clusters (b) or alone (c).

The incorporation of IBU@MSN-SA-Ca nanoparticles into the PVDF fibres was optimised to maximise nanoparticle loading while maintaining acceptable formulation processability and the overall properties of the final composite scaffold. Therefore, increasing concentrations of IBU@MSN-SA-Ca, particularly 1% wt/vol, 5% wt/vol, and 10% wt/vol, were tested. As shown in Fig. S9, S10 and 9, aligned fibres were obtained at all concentrations tested using similar ESP parameters (drum speed, spinneret to collector distance, voltage), except for the needle size. For PVDF_MSN5_IBU (5% wt/vol) and PVDF_MSN10_IBU (10% wt/vol), it was necessary to modify the diameter from 22 G to 16 G due to the formulation's increased viscosity, which restricted flow through narrower capillaries. PVDF_MSN1_IBU and PVDF_MSN5_IBU fibres were smooth, defect-free and exhibited comparable diameters of 250 ± 78 nm and 265 ± 96 nm, respectively. IBU@MSN-SA-Ca nanoparticles were well dispersed within the polymeric matrix, appearing on the surface of the electrospun fibers either as small clusters (Fig. 9b and S9b) or as individual particles (Fig. 9c and S9c). Additionally, the localised swellings observed along the fibers suggest that some nanoparticles may also be embedded within the fiber core. In contrast, PVFD_MSN10_IBU fibres retained a preferential orientation (Fig. S10a), but displayed numerous defects, large nanoparticle clusters (Fig. S10b), and fiber bundles (Fig. S10c). In addition, the measured diameter was 277 ± 157 nm, indicating greater variability compared to the other samples. This variability can be attributed to the presence of nanoparticles, which, at higher concentrations in the spinning suspension, may interfere with the formation of a stable Taylor cone, leading to the deposition of fibers with heterogeneous diameters. However, anisotropic scaffolds composed of aligned nanofibers with diameters ranging from 150 to 750 nm have been proposed in the literature for the restoration of various tissues—including bone, nerve and myocardium^14,79,80^—due to their ability to mimic the oriented morphology of the ECM of these tissues. This structural mimicry offers contact guidance that support cell spreading, differentiation, and the formation of intracellular structures resembling those found in native tissue. Accordingly, all tested scaffolds demonstrate suitability for tissue regeneration applications.

To confirm the retention of piezoelectricity, the effect of nanoparticles loading into the ESP formulation on the formation of the β phase was also investigated. As indicated by the values calculated by FTIR-ATR (Tables 2 and S2), the β phase content remained very high (>92%) when the amount of IBU@MSN-SA-Ca was relatively limited, as for PVDF_MSN1_IBU and PVDF_MSN5_IBU samples. However, incorporating higher nanoparticle concentrations into the PVDF solution, as seen in PVDF_MSN10_IBU samples, led to a reduction in β phase content to approximately 85%. Among the isomorphs of PVDF, the most stable form is the *cis*–*trans* α phase, which naturally forms during crystallisation but lacks piezoelectric properties due to its apolar conformation.^81^ To induce the formation of β phase and impart piezo responsiveness to PVDF, mechanical stretching and electrical poling are commonly employed to align molecular dipoles. The use of ESP with a high-speed rotating collector inherently applies electromechanical stress during fibre formation, promoting the β phase presence in PVDF. Depending on the processing parameters, the β phase has been reported to range from 70% up to 90%.^82^ In this study, all tested samples showed a β phase content comparable to the highest values reported in the literature. This outcome can be attributed to the high voltage used during processing and the strong dipole moment of the solvent mixture, particularly of DMSO, which facilitates polymeric chain reorientations.^83^ Notably, the incorporation of nanoparticles did not hinder PVDF molecular stretching when used at limited concentrations, such as 1% and 5% wt/vol in the formulations.

**Table 2:** Crystalline structure, piezoelectric and mechanical properties of PVDF_MSN5_IBU scaffold^a^

		PVDF_MSN5_IBU
Crystalline structure	*X*_c_ (%)	52 ± 1
	α phase (%)	7 ± 2
	β phase (%)	93 ± 3
	γ phase (%)	n.d.
Piezoelectric properties	*d*_33_ (pm V^−1^)	−7 ± 2
Mechanical properties	*E* (MPa)	40 ± 7
	Deformation (%)	62 ± 5
	Ultimate strength (MPa)	6 ± 1

a n.d.: the γ phase was not detected within the measurement uncertainty.

Firstly, prolonged deposition experiments were conducted with formulations containing the highest concentration of nanoparticles. Significant challenges arose in processing the PVDF_MSN10_IBU formulation: the Taylor cone exhibited extreme instability, frequent needle clogging occurred, and rapid nanoparticle sedimentation was observed in the reservoir. To improve processability, the IBU@MSN-SA-Ca load was reduced incrementally. Before reducing it to 5% wt/vol, an intermediate concentration of 7.5% wt/vol was tested. Although this adjustment mitigated some issues compared to PVDF_MSN10_IBU, the 7.5% wt/vol concentration was still unsuitable for producing thick scaffolds due to persistent processing issues. Consequently, samples with this intermediate concentration were discarded without further characterisation. In contrast, the PVDF_MSN5_IBU suspension demonstrated excellent processability, remaining stable for up to three hours, resulting in membranes that could be easily detached from the substrate and handled without difficulty. Thus, the 5% wt/vol concentration was identified as the optimal IBU@MSN-SA-Ca nanoparticles load for the PVDF formulation and was chosen for subsequent characterisation and drug release experiments. The properties of PVDF_MSN5_IBU, including crystallinity, piezoelectricity, and mechanical performance, are detailed in Table 2. In addition to the β phase, the total crystallinity (*X*_c_) of PVDF_MSN5_IBU fibres is high (52%), comparable to the best values of *X*_c_ reported in the literature.^82–84^ The nanoparticles not only did not affect β phase formation but also did not influence crystallization. Thanks to the high β phase content and crystallinity, the composite scaffold demonstrated piezoelectric behaviour, with a *d*_33_ coefficient of −7 pm V^−1^. Notably, scaffolds with similar piezoelectricity have been reported to successfully support the activity of various cell types, such as cardiomyocytes^85^ and Schwann cells.^86^ Additionally, the mechanical properties of PVDF_MSN5_IBU membranes (Table 2 and Fig. S11) closely resemble those reported for aligned PVDF scaffolds used also in tissue regeneration applications,^14,87,88^ confirming that the nanoparticles did not compromise the overall fibrous structure. As reported in Fig. S12, thermogravimetric analysis on PVDF_MSN5_IBU showed at 1000 °C a residual weight of 9.5%, ascribed to silica residues not thermally decomposed. Based on the residual weight of 59% wt measured at 1000 °C for the IBU@MSN-SA-Ca nanoparticles alone, and considering that the PVDF scaffold exhibits no residual weight as expected, the nanoparticle content incorporated into the electrospun scaffold was calculated to be 16% wt. Given the ibuprofen loading in the nanoparticles (13.5% wt), the ibuprofen content in the composite scaffold was determined to be 2.2% wt. This value is consistent with the theoretical value of 2.9% wt, estimated based on nanoparticles added to the PVDF solution for electrospinning (*i.e.*, 5% wt/vol of IBU@MSN-SA-Ca into a 18% wt/vol PVDF solution in ACE : DMSO), assuming complete evaporation of the solvent mixture. The ibuprofen release profile from the PVDF_MSN5_IBU scaffold is reported in Fig. S13. Following an initial burst release of the drug within the first 72 hours, the scaffold exhibited a sustained release profile, with 40% of the total ibuprofen released over a 28-day period.

#### 3.3.1 Biological tests on the composite scaffold

**Fig. 10:**
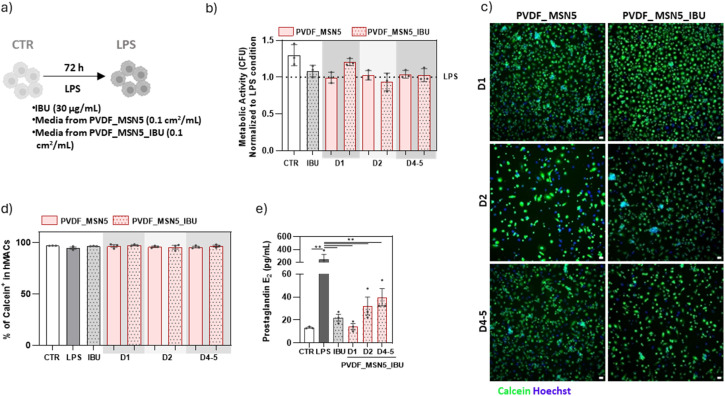
Evaluation of biocompatibility and anti-inflammatory properties of the components released by PVDF_MSN5 and PVDF_MSN5_IBU on hMACs. (a) hMACs were stimulated with LPS, either in the presence or absence of IBU, or media released by PVDF_MSN5 and PVDF_MSN5_IBU during day 1 (D1), day 2 (D2) and day 4–5 (D4–5), during 72 h. hMACs cultured in the absence of LPS were used as control (CRT). (b) Evaluation of hMAC metabolic activity. Results are normalized to LPS condition (*n* = 3 patients/group). (c) and (d) Representative images (c) and respective quantification (d) of hMAC viability. hMACs were stained with calcein (green) and the nuclei stained with Hoechst (blue). Scale bars = 100 μm (*n* = 4 patients/group). (e) Quantification of secreted PGE_2_ (pg mL^−1^) by hMAC (*n* = 4 patients/group). Data show mean ± SEM. For statistical analysis a parametric multiple comparison evaluation through paired one-way ANOVA test was performed to assess differences between groups. Statistical significance is indicated as *p < 0.05, **p < 0.01, ***p < 0.001 and ****p < 0.0001.

Building upon the promising results obtained with the IBU@MSN-SA-Ca nanoparticles, we next sought to evaluate the performance of the PVDF_MSN5_IBU scaffold under comparable experimental conditions. We used a PVDF scaffold containing 5% wt/vol MSN-SA-Ca (named PVDF_MSN5) to compare the results. To more closely mimic the *in vivo* drug release conditions following scaffold implantation, an indirect assay was conducted. For that, the components released from a scaffold concentration of 0.1 cm^2^ mL^−1^ during day 1 (D1), day 2 (D2) and from day 4 to 5 (D4–5) were collected and subsequently analysed for both biocompatibility and anti-inflammatory efficacy on hMACs (Fig. 10a).

The media released from the scaffold did not show any cytotoxic effects at any of the tested time points, highlighting its safety for therapeutic application, as demonstrated by metabolic activity and calcein^+^ cells (Fig. 10b and d). Metabolic activity assays and calcein^+^ cell quantification confirmed that all tested conditions supported cell viability and were well-tolerated over time. In terms of anti-inflammatory efficacy, a significant reduction of PGE_2_ levels was observed at every time point assessed (Fig. 10e). A progressive increase in average PGE_2_ was observed at extended time points, likely due to the progressive reduction in IBU release following the initial phase.

These results demonstrate that the nanoparticles retain their anti-inflammatory effect once incorporated into the scaffold. Given its excellent cytocompatibility and demonstrated *in vitro* anti-inflammatory efficacy, the biomimetic scaffold exhibits strong potential for attenuating inflammation while providing structural support for the regeneration of injured tissues.

#### 3.3.2 US stimulation on the composite scaffold

**Fig. 11:**
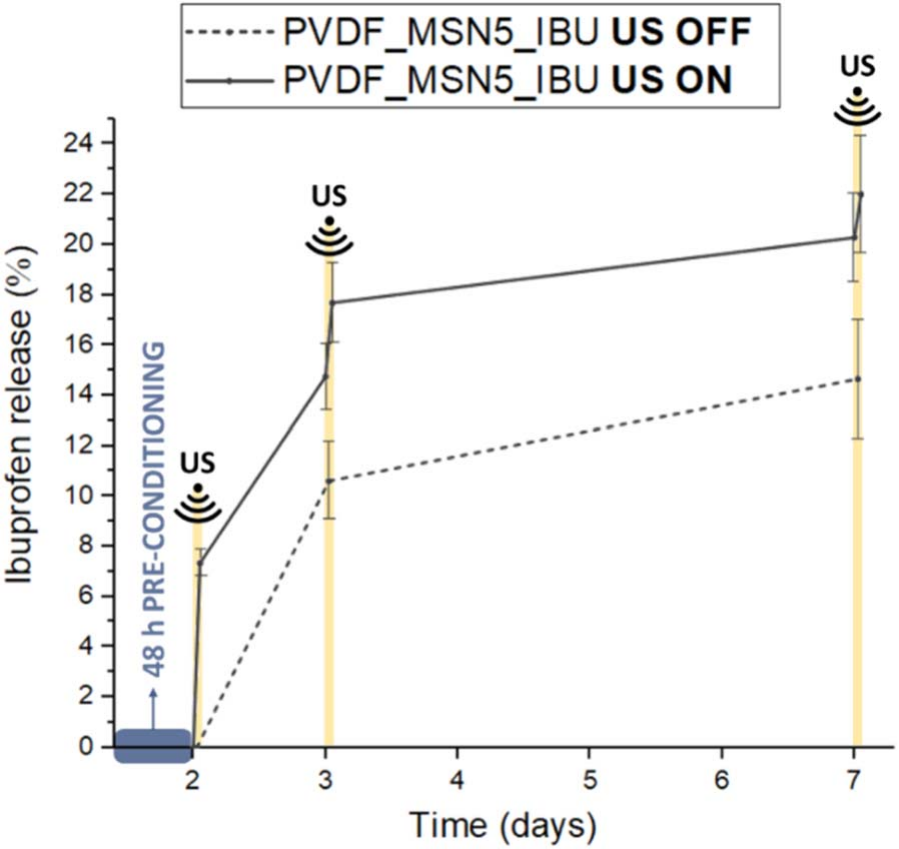
Drug release from PVDF_MSN5_IBU scaffold in the absence of US stimulation (dashed line) and upon optimized US stimulation applied at day 2, day 3 and day 7 (solid line).

As the therapeutic efficacy of the scaffold progressively diminishes over time, the feasibility of regulating IBU release from the composite system through repeated US stimulation, even at a later stage, was investigated. For that, PVDF_MSN5_IBU was pre-conditioned and subjected to US stimulations at day 2, 3 and 7 (Fig. 11). The US parameters used for the scaffold stimulation were those optimised for the nanoparticles alone, but preliminary tests revealed that extending the stimulation duration to 5 minutes increased ibuprofen release. The results of the drug release test on PVDF_MSN5_IBU upon US stimulation are reported in Fig. 11. Upon a single US application, 7% of the loaded ibuprofen was released from the scaffold. After 24 h, the second US stimulation induced the release of 3% of the drug, while the third stimulation, applied 5 days later, resulted in an additional 1.5% ibuprofen release.

The results showed that the US-responsivity of the nanoparticles was retained after their incorporation into the electrospun structure. As expected, due to the shielding effect of the PVDF matrix, which attenuates the exposure of the nanoparticles to US and slows drug diffusion, a slight reduction in US-responsiveness was observed compared to free IBU@MSN-SA-Ca. Overall, these findings demonstrate the capability of PVDF_MSN5_IBU to provide US-enhanced ibuprofen release, even after multiple stimulations applied up to seven days post-immersion. Building on its biocompatibility and capacity for drug release upon safe and non-invasive stimulation, the developed scaffold represents a promising strategy for providing structural support and enabling spatiotemporally controlled modulation of inflammation, thereby promoting the functional repair of damaged tissues.

## 4. Conclusions

This study reports the development of ibuprofen-loaded mesoporous silica nanoparticles functionalized with an ultrasound (US)-responsive alginate coating (IBU@MSN-SA-Ca), enabling precise modulation of drug release timing and dosage. Alginate grafting was optimised to preserve nanoparticle morphology while enhancing polymer packing upon crosslinking, thereby improving surface capping efficiency and drug retention.

*In vitro* biocompatibility was confirmed using human macrophages at concentrations up to 1 mg mL^−1^. Anti-inflammatory efficacy was evaluated *via* PGE_2_ inhibition both in direct mode, contacting the human macrophages with nanoparticle suspension (1 mg mL^−1^ and 0.1 mg mL^−1^), and in indirect mode, *i.e.*, contacting the cells with the drug release extracts from nanoparticle suspensions (1 mg mL^−1^ and 0.1 mg mL^−1^) at different timepoints. Both approaches demonstrated significant PGE_2_ suppression, with sustained activity observed through days 4–5.

A systematic investigation of US stimulation parameters identified optimal conditions that minimised thermal effects from energy dissipation while maximising controlled drug release. Medium-frequency and high-intensity settings proved most effective, with stimulation duration showing limited influence. US-triggered release resulted in a dose-dependent reduction of PGE_2_ levels, achieving a two-fold enhancement in anti-inflammatory activity at nanoparticle concentrations ≥0.05 mg mL^−1^.

The developed nanoparticles were incorporated into an electrospun piezoelectric PVDF scaffold, harnessing the synergistic effects of its biomimetic architecture—which enhances cell adhesion and proliferation—and US-responsive ibuprofen release. This integration yielded a multifunctional platform designed to comprehensively support tissue regeneration. Scaffold fabrication was optimised to maximise nanoparticle incorporation while preserving biomimetic features. The resulting composite scaffold presented aligned nanofibers, along with suitable piezoelectric and mechanical properties to support the regeneration of aligned electroactive tissues. *In vitro* evaluations confirmed the scaffold's excellent biocompatibility and its effective anti-inflammatory properties. Moreover, US-responsiveness of the nanoparticles was retained after their incorporation into the scaffold, which exhibited enhanced ibuprofen release in response to multiple US stimulation cycles applied up to seven days post-immersion.

By integrating stimuli-responsive materials with electrospinning technology, this study introduces a novel strategy to address the complex dynamics of tissue regeneration. The synergistic interaction between US stimulation and drug-eluting piezoelectric constructs offers promising avenues for future research into functional tissue remodelling. Indeed, electrical signals generated through US-induced mechanical stimulation of piezoelectric components have been shown to promote tissue regeneration,^17,89,90^ while US-mediated permeabilization of biological barriers is expected to enhance therapeutic delivery and efficacy.^91^ These complementary mechanisms are anticipated to further enhance the performance of the developed constructs, which—by mimicking the electromechanical properties of native tissue and enabling localised, on-demand release of anti-inflammatory agents—provide a multifunctional platform to support and promote the functional repair of damaged tissues.

## Author contributions

A. B. M.: conceptualization, formal analysis, investigation, methodology, validation, visualization, writing original draft; J. B.: conceptualization, formal analysis, investigation, methodology, validation, visualization, writing original draft; G. M.: conceptualization, methodology; writing – review and editing; E. M.: investigation, formal analysis; S. F. G.: investigation, formal analysis, methodology; E. S.: investigation, formal analysis, validation, visualization, writing original draft; R. G: investigation, formal analysis; D. N.: supervision, writing – review and editing; S. S.: methodology (ultrasound setup); A. C.: methodology (ultrasound setup); S. F.: conceptualization, supervision, funding acquisition, project administration, writing – review and editing; C. V.-B.: conceptualization, supervision, funding acquisition, project administration, writing – review and editing.

## Conflicts of interest

There are no conflicts to declare.

## Supplementary Material

RA-015-D5RA05217C-s001

## Data Availability

The data supporting this article are included within the main text or the SI. Additional data are available from the authors upon request. See DOI: https://doi.org/10.1039/d5ra05217c.
