# Supplementary material for: Piezoelectric electrospun scaffold incorporating ibuprofen loaded ultrasound-responsive mesoporous silica nanoparticles for tissue regeneration
Source: RSC Adv. 2025 Sep 17;15(40):33868–83. doi: 10.1039/d5ra05217c (PMC12442029; doi:10.1039/d5ra05217c)
Supplement: RA-015-D5RA05217C-s001 [file RA-015-D5RA05217C-s001.pdf]

Supplementary Information (SI) for RSC Advances.
This journal is © The Royal Society of Chemistry 2025

SUPPLEMENTARY INFORMATION

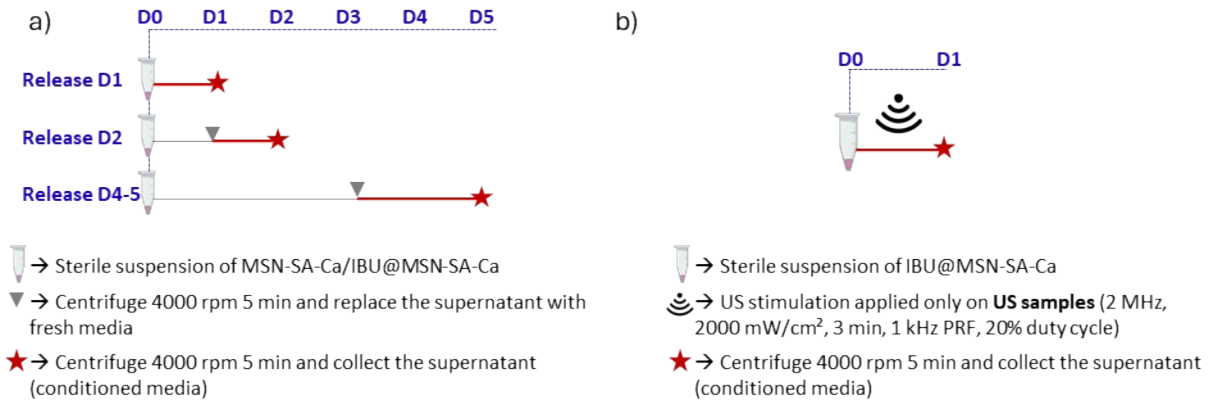

Figure S1 a) Methodology of in vitro indirect biocompatibility and efficacy assay on the nanoparticles and on the scaffold. **b)** Methodology of in vitro assay of the anti-inflammatory efficacy of the nanoparticles upon US stimulation.

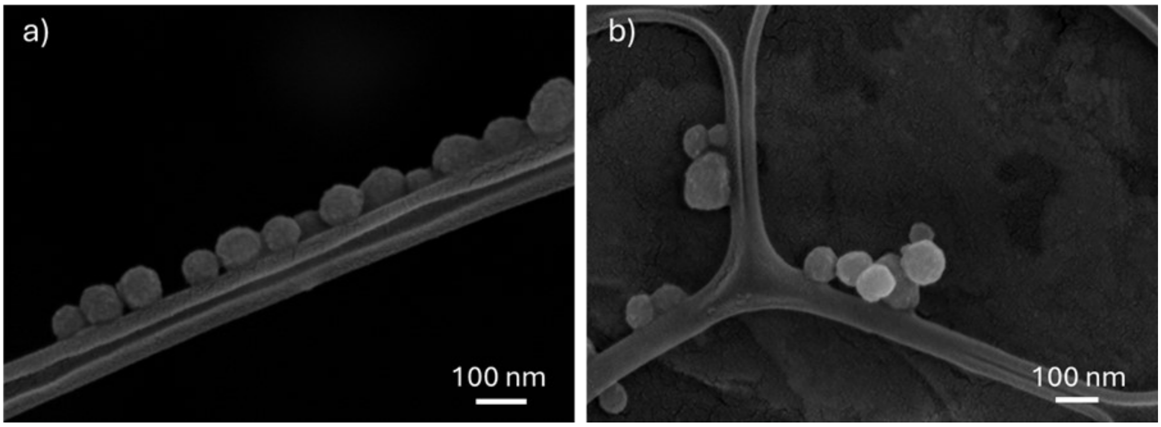

Figure S2 FESEM images of **a)** MSN after calcination **b)** MSN-NH_2.

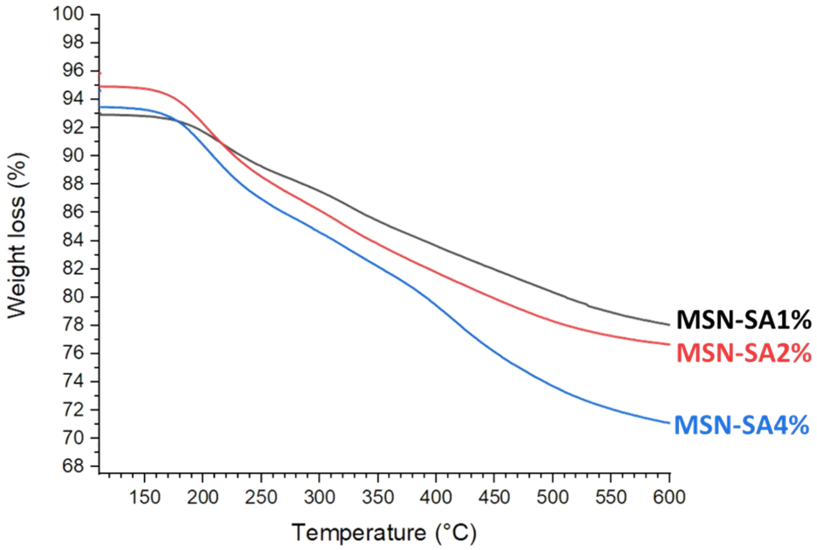

Figure S3 TGA of MSN-SA grafted with different concentrations of sodium alginate: 1% wt/vol (MSN-SA1%, black line), 2% wt/vol (MSN-SA2%, red) and 4% wt/vol SA (MSN-SA4%, blue).

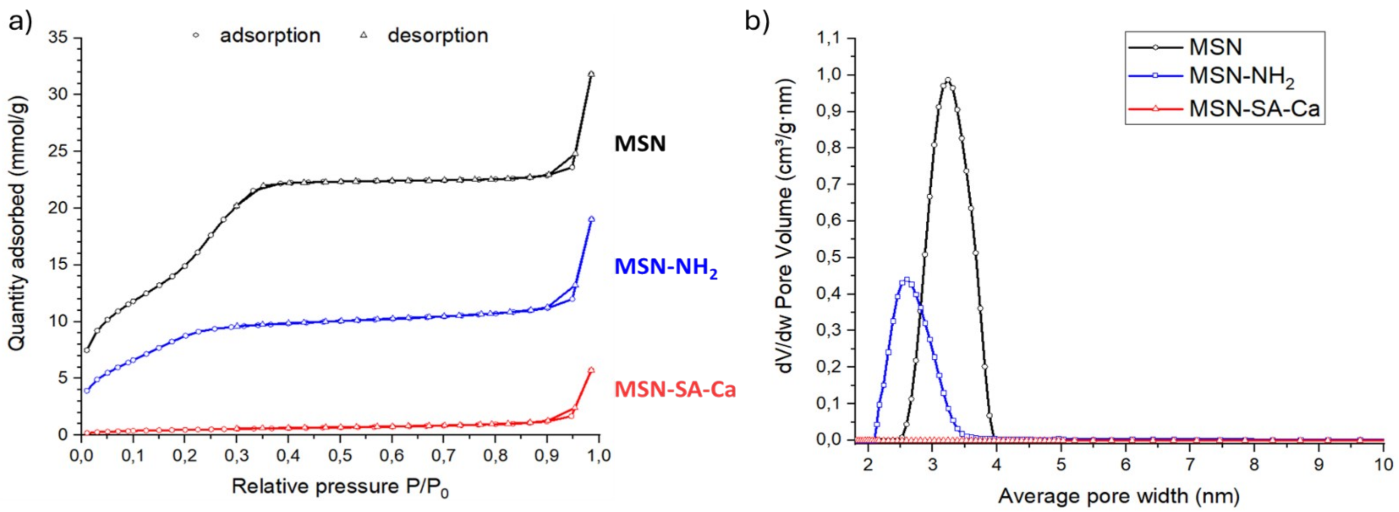

Figure S4 a) N_2 adsorption-desorption isotherms and **b)** pore size distributions of MSN (black curve), MSN-NH_2 (blue), and MSN-SA-Ca (red).

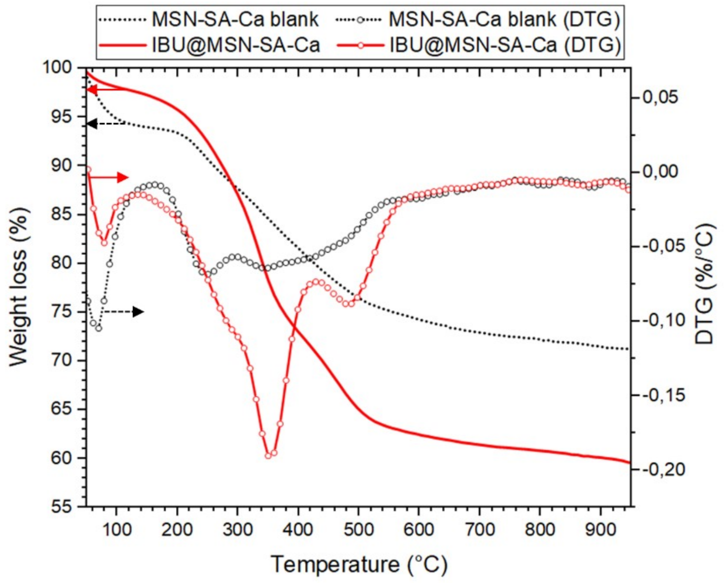

Figure S5 TGA and DTG curves of MSN-SA-Ca (dashed lines) and IBU@MSN-SA-Ca (solid lines).

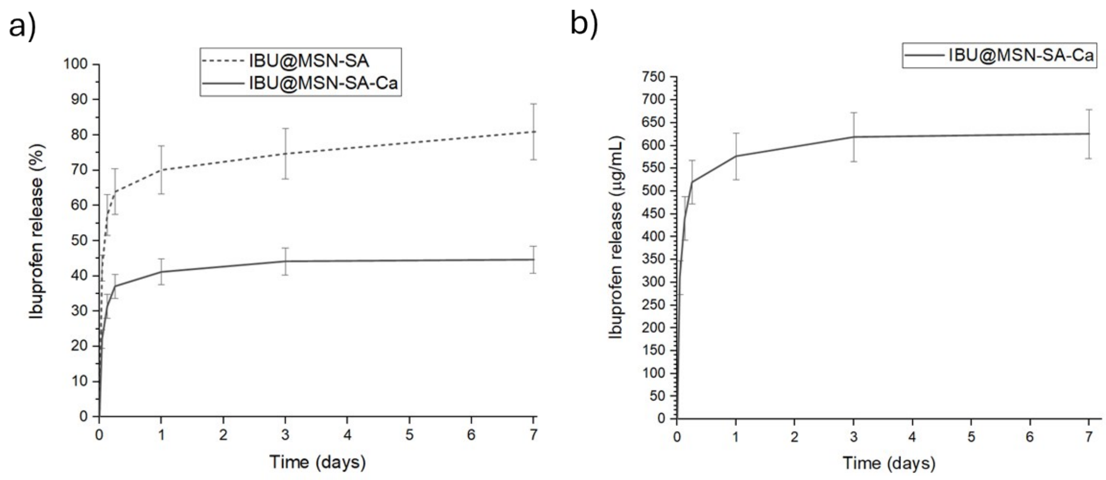

Figure S6 a) Ibuprofen release (% on the total IBU loading) from the nanocarriers before (IBU@MSN-SA) and after crosslinking (IBU@MSN-SA-Ca), in the absence of US stimulation; **b)** Ibuprofen release (µg/mL) from a 10 mg/mL suspension of IBU@MSN-SA-Ca, in the absence of US stimulation.

Table S1 US stimulation parameters used in the study.

Experiment	Frequency (kHz)	Intensity (mW/cm²)	Pulse Repetition Frequency (kHz)	Duty Cycle (%)	Stimulation duration (min)
A1	38	500	1	20	3
A2	2000	500	1	20	3
A3	5000	500	1	20	3
B1	2000	250	1	20	3
B2	2000	500	1	20	3
B3	2000	1000	1	20	3
B4	2000	2000	1	20	3
C1	2000	2000	1	20	5

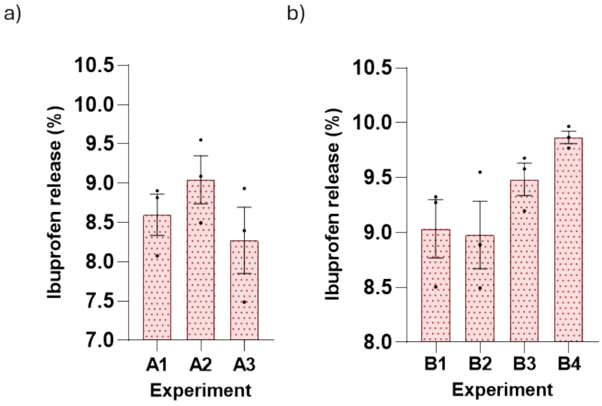

Figure S7 Ibuprofen release from IBU@MSN-SA-Ca upon US stimulation. **a)** Effect of varying frequency at a fixed intensity of 500 mW/cm^2 (experiments A); **b)** Effect of varying intensity at a fixed frequency of 2 MHz (experiments B). Data show mean ± SEM.

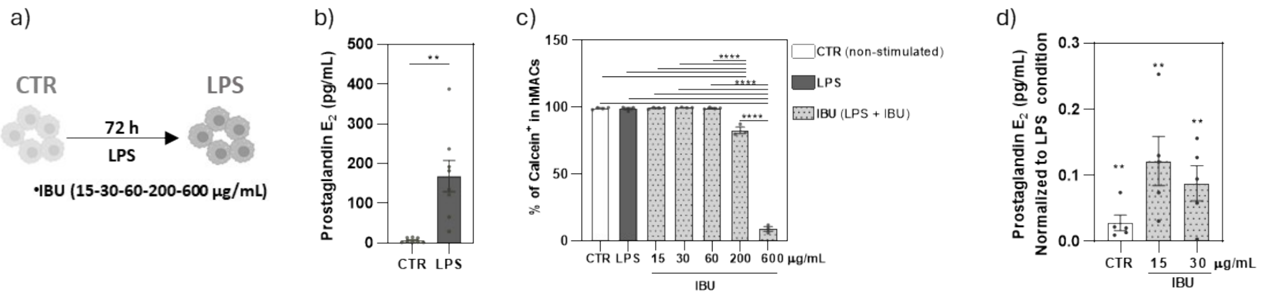

Figure S8 Determination of the therapeutic window of IBU on hMACs in vitro. **a)** hMACs were stimulated with LPS, either in the presence or absence of IBU, during 72h. **b)** Quantification of secreted PGE_2 (pg/mL) by hMACs when maintained in non-stimulated conditions (CTR) or stimulated with LPS (n=5 patients/group). **c)** Quantification of hMAC viability, as determined by % of $Calcein^+$ cells, when maintained as CTR, LPS or stimulated with LPS in the presence of increasing concentrations of IBU for 72 h (n≥3 patients/group). **d)** Quantification of secreted PGE_2 (pg/mL) by hMAC maintained as CTR or stimulated with LPS in the presence of 15 μg/mL and 30 μg/mL of IBU. Data were normalized to LPS condition (n=5 patients/group). Data show mean ± SEM. For statistical analysis a parametric multiple comparison evaluation through paired one-way ANOVA test was performed to assess differences between groups. Statistical significance is indicated as $*p < 0.05$, $**p < 0.01$, $***p < 0.001$ and $****p < 0.0001$.

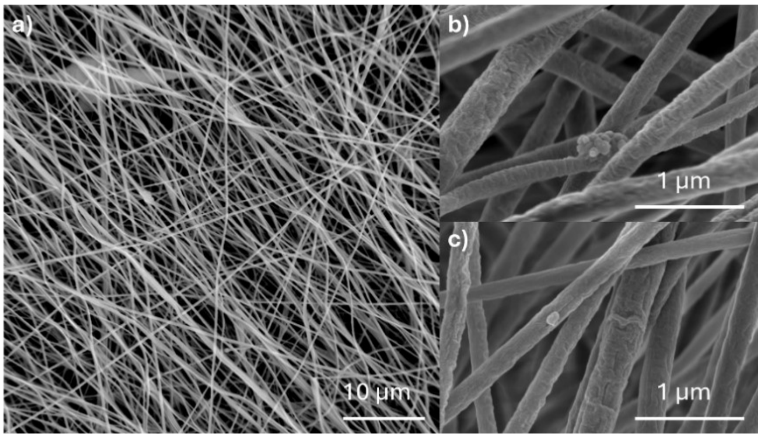

Figure S9 a) FESEM image of PVDF_MSN1_IBU; details of IBU@MSN-SA-Ca in the fibres in small clusters **(b)** or alone **(c)**.

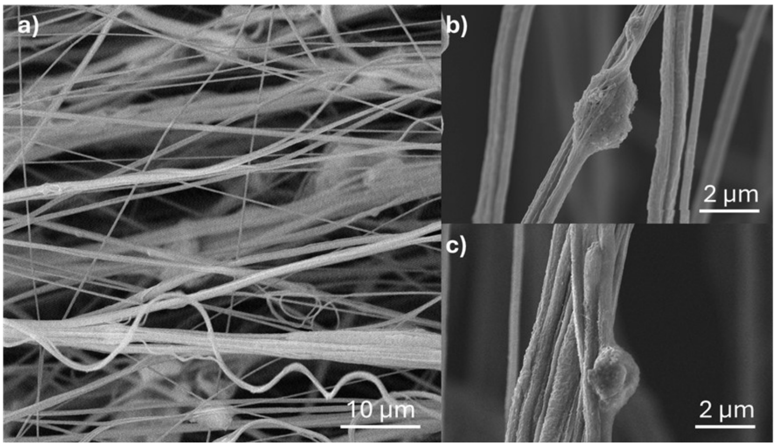

Figure S10 a) FESEM image of PVDF_MSN10_IBU; details of IBU@MSN-SA-Ca in the fibres in large clusters **(b)** and fibre bundles **(c)**.

Table S2 Diameter and relative ß phase content of PVDF_MSN1_IBU and PVDF_MSN10_IBU scaffolds.

Sample	Diameter (nm)	ß phase (%)
**PVDF_MSN1_IBU**	250±80	92±2
**PVDF_MSN10_IBU**	280±160	85±3

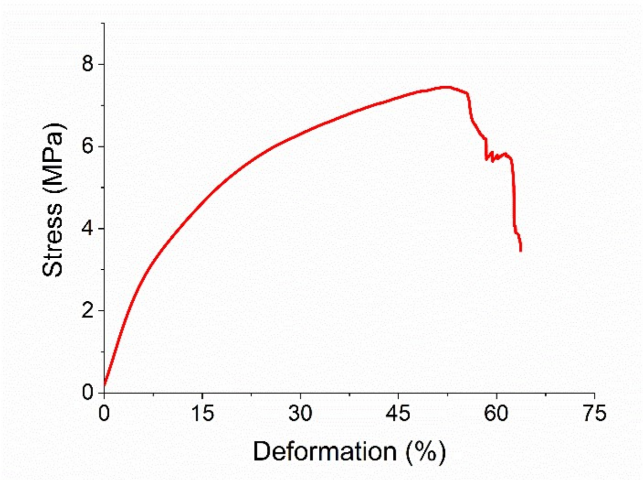

Figure S11 Representative stress deformation curve for PVDF_MSN5_IBU membrane.

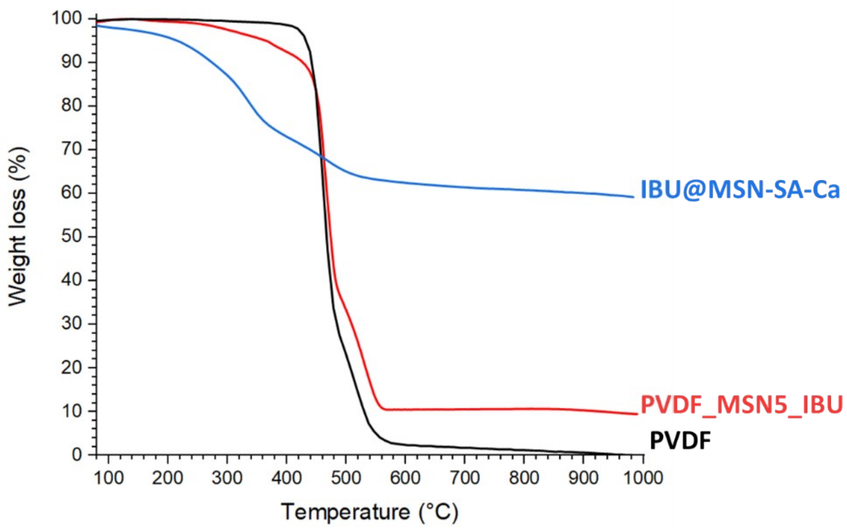

Figure S12 TGA analysis on pure PVDF scaffold (black curve), IBU@MSN-SA-Ca (blue), and PVDF_MSN5_IBU composite scaffold (red).

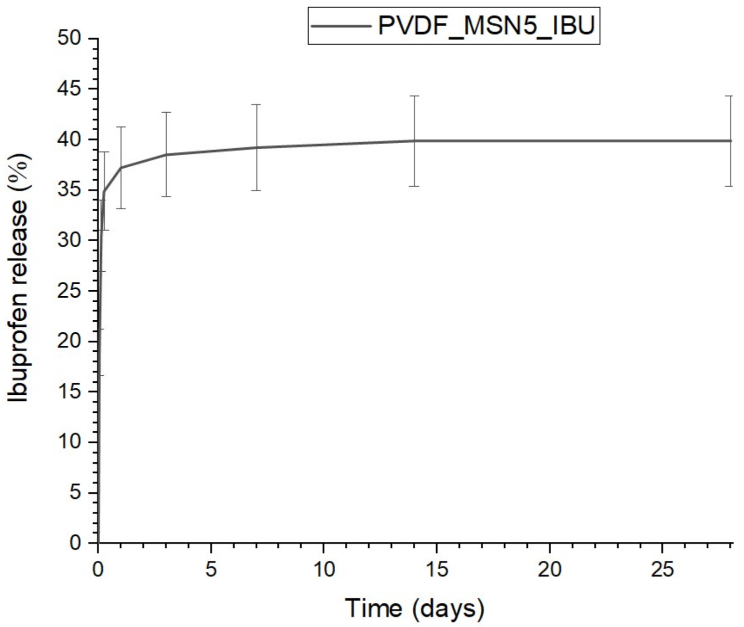

Figure S13 Drug release from the PVDF_MSN5_IBU scaffold in the absence of US stimulation.
